# Perceived relevance of Planetary Health for Medical Students in Italy: Results from a mixed-methods analysis

**DOI:** 10.1016/j.puhip.2025.100607

**Published:** 2025-03-25

**Authors:** L. Nachira, P. Arcaro, F. Pattavina, E. Campo, C. Castagna, R. Frasso, C. Cadeddu, S. Bruno

**Affiliations:** a Section of Hygiene, Department of Life Sciences and Public Health, Università Cattolica del Sacro Cuore, Largo F. Vito 1, 00168, Rome RM, Italy; b Women, Children and Public Health Sciences Department, Fondazione Policlinico Universitario A. Gemelli IRCCS, Largo A. Gemelli 8, 00168, Rome RM, Italy; c Jefferson College of Population Health, Thomas Jefferson University, 901 Walnut St, Philadelphia, PA 19103, USA; d Asano-Gonnella Center for Research in Medical Education and Health Care, Sidney Kimmel Medical College, Thomas Jefferson University, 1025 Walnut Street, Philadelphia, PA 19107, USA; e Erasmus School of Health Policy and Management, Erasmus University of Rotterdam, Burgemeester Oudlaan 50, 3062 PA, Rotterdam, the Netherlands

**Keywords:** Planetary health education, Medical students, Elective course, Medical curriculum, Medical training

## Abstract

**Objectives:**

To address the growing hazards to human health caused by the anthropogenic environmental catastrophe, academic curricula at all levels and disciplines, particularly medical education, should incorporate Planetary Health Education. This study aims to examine medical students' expectations and feedback on a Planetary Health elective in an Italian University.

**Study design:**

Cross-sectional study, employing a convergent parallel mixed-methods design.

**Methods:**

Attendees were invited to take part in a mixed-methods written survey, integrating both quantitative and qualitative methodologies. A statistical and a thematic analysis were conducted to assess responses to questions of a quantitative and qualitative nature, respectively.

**Results:**

Interest in the climate crisis was the main reason for participating in the course (52·7 %), followed by interest in a multidisciplinary approach to health (48·6 %), and the focus on the relationship between human, animal and environmental health (48·6 %). Five themes emerged from the thematic analysis: relationship between human health and the environment, international health emergencies, characteristics and role of health systems, broadening of knowledge and views, positive professional impact of the course.

**Conclusion:**

The survey participants showed a deep understanding of the link between environmental conservation and the safeguarding of human health, suggesting that the next generation of medical practitioners could make a significant difference in healthcare and beyond.

## Abbreviations

CFMS HEART: Canadian Federation of Medical Students Health and Environment Adaptive Response Task Force
ES: Educational Session
IFMSA: International Federation of Medical Student Associations
PH: Planetary Health
PHA: Planetary Health Alliance
PHE: Planetary Health Education
PHEC: Planetary Health Educational Competencies
PHRC: Planetary Health Report Card
UCSC: Università Cattolica del Sacro Cuore

## 1 Introduction

### 1.1 Background

Human health is increasingly threatened by the anthropogenic environmental crises, which encompasses climate change and the transgression of other planetary boundaries ^2,3^and is disproportionately affecting the poorest countries and most vulnerable communities, exacerbating health inequalities [3].

Planetary Health (PH) emerges as “a solutions-oriented, transdisciplinary field and social movement focused on analyzing and addressing the impacts of human disruptions to Earth's natural systems on human health and all life on Earth” [1,2]. To obtain significant and long-lasting effects, Planetary Health Education (PHE) should be integrated into academic curricula across all levels and disciplines [3], especially in medical education [4,5]. Several medical schools worldwide featured student-led or informal initiatives on these themes [4,6], but only 14·7 % have currently included climate change and health topics within medical curricula in 2019, and 11 % had offered formal education on air pollution and health in 2020 [6].

### 1.2 Aim

The purpose of this study, in the context of an elective class on PH among medical students attending Università Cattolica del Sacro Cuore (UCSC), in Rome, Italy, was to (I) assess expectations and prior knowledge about PH; (II) evaluate satisfaction with the course; (III) gather suggestions to improve it, and opinions on the relevance of the structural integration of PHE into medical curriculum. This work seeks to provide valuable insights into medical students’ perspectives about Planetary Health, that can help design tailored PH courses to be implemented in medical schools.

## 2 Methods

### 2.1 Study design, population, and setting

This cross-sectional study employed a convergent parallel mixed-methods design, and was reported according to the Strengthening the Reporting of Observational Studies in Epidemiology (STROBE) [7] and COnsolidated criteria for REporting Qualitative research (COREQ) guidelines [8].

A survey was conducted during a PH elective offered to fifth-year medical students at UCSC, resulting in a convenience sample. Four educational sessions (ESs), each lasting 2 h, were led by expert lecturers between October–December 2022; a prospect of the lessons and the lecturers is presented in Table 1. The experts and topics were chosen to cover general PH issues, since this was the first time students received formal education on PH.

**Table 1:** Overview of the course: title, content and lecturer's background for each educational session (ES).

Titles of the ES	Content	Lecturer's background
Planetary Health: role and responsibilities of Public Health (ES1)	It provided an introduction to planetary health, highlighting its relationship with public health	Medical doctor, Public Health professor
One Health, EcoHealth and Planetary Health approaches (ES2)	It provided a comparison of different approaches to studying the relationship between human and environmental health	Natural Scientist
Planetary Health: the role of health response in population emergencies (ES3)	It illustrated the organizational aspects of managing climate-related health emergencies	Medical doctor, Public Health specialist
The Role of Development Cooperation in Countries with Limited Resources, and its interconnection with Planetary Health (ES4)	It described field experiences in managing climate-induced migration	Medical doctor, specialist in occupational medicine

All ESs consisted of an 80-min didactic lecture followed by approximately 40 min of discussion, in which students were invited to present their reflections and ask questions related to the topics of the day. They were held in person, except for the second one, during which the lecturer participated remotely while students gathered in a physical classroom.

### 2.2 Data collection

Three different voluntary and anonymous questionnaires were administered at separate time points: one before the course began (pre-course), one following each ES (post-ES, n = 4), one at the completion of the course (post-course). The questionnaires were based on validated instruments used in similar studies [9,10]; the questions were the same as in those studies, with only minor modifications in three questions to reflect the different topics of this course (e.g. the title of the ESs, and the course-specific topics of interest). Questionnaires were administered, and answers analyzed, in Italian. Questions and answers were then translated into English for dissemination purposes.

The pre-course questionnaire included multiple-answer questions investigating motivations for enrolling in the class, and four open-ended questions designed to explore students’ baseline understanding of PH, expectations for the course, topic preferences, and thoughts on how PH issues will impact their future careers. The post-ES and post-course questionnaires included linear scale questions with scores ranging from 1 to 4, investigating satisfaction with various aspects of each ES and the whole elective, respectively. Furthermore, the post-course questionnaire contained an open-ended question gathering suggestions and comments.

### 2.3 Data analysis

Data were recorded into an Excel 2016 database. Categorical variables were converted into discrete numerical variables. To protect respondents’ anonymity and due to variations in participation across the different data collection phases, data pairing was not feasible. A directed content analysis of the written answers to the open-ended question section was performed by three MDs, Public Health resident doctors (EC, LN, PA). This analysis was performed in Italian. Free text was reported into an Excel database, and a thematic analysis was performed after developing a codebook based on the questions posed and on a preliminary analysis of a random sample of answers. This tool was refined during several meetings among the research team (CCas, EC, LN, PA) to better adapt to the answers through discussion and literature research. Subsequently, all answers were categorized according to the final version of the codebook, resolving coding discrepancies through discussion within the team (CCas, EC, LN, PA). The phase of data collection, coding and analysis was guided by a qualitative methodologist with more than 20 years of experience (RF). The quantitative and qualitative findings were integrated at the interpretive level, using a weaving approach [11].

### 2.4 Statistical analysis of the quantitative data

Quantitative variables were reported as absolute frequency and percentage. For quantitative variables, medians, interquartile ranges (IQR), means and standard deviations (SD) were calculated. A *t*-test and ANOVA analysis were performed to determine the correlation between satisfaction for each ES, considering statistically significant *p*-values <0·05. All analyses were performed using Stata 16.1 software (StataCorp LLC, College Station, TX, USA, 2019).

## 3 Results

Of the 271 fifth-year medical students, 80 enrolled in the elective course, of which 74 completed the pre-course questionnaire and 33 the post-course one. Responses rates varied across ES: 70 students completed the post-ES questionnaire for ES1; 44 for ES2; 46 for ES3; and 34 for ES4.

Median age of respondents was 23 (IQR = 22–24), and the percentages of respondents identifying as male and female were 50 % and 48·6 %, respectively, with 1·4 % preferring not to disclose their gender; at UCSC, approximately 60 % of the total fifth-year medical students are female and 40 % are male.

### 3.1 Pre-course findings

#### 3.1.1 Quantitative findings

Pre-course questionnaires showed that interest in the climate crisis was the main reason for enrolling (52·7 %), followed by the multidisciplinary approach to health (48·6 %), and the focus on the relationship between human, animal and environmental health (48·6 %). Interest in Public Health (43·2 %), health response to emergencies (36·5 %), and human-nature interactions (32·4 %) were also common motivations. A smaller number indicated interest in PH (29·7 %) and the willingness to complement the undergraduate curriculum (29·7 %).

#### 3.1.2 Qualitative findings

The 4 open-ended questions in the pre-course questionnaires were answered by 71 participants. Responses to open-ended questions varied in structure, with some respondents sharing lists of words and other longer narratives. Analysis of the pre-course responses yielded five themes.

- Relationship between human health and the environment
- International health emergencies
- Characteristics and role of health systems
- Broadening of knowledge and views
- Positive professional impact of the course

**Fig. 1:**
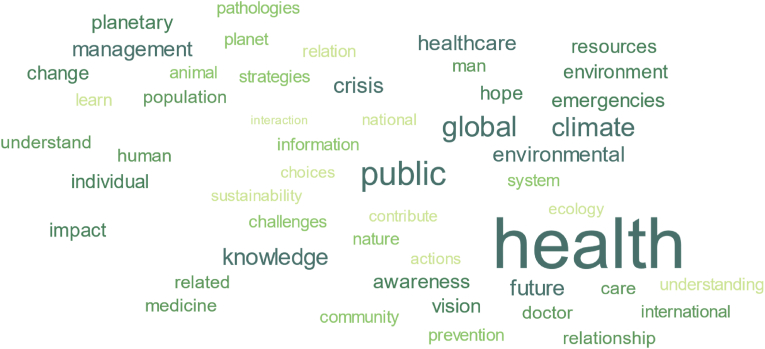
Word cloud of the 50 most frequent words from the students' written answers to pre-course questions.

Below each theme is described briefly and supported by selected participant responses to pre-course questionnaires, translated to English. Fig. 1 provides a word cloud of the 50 most frequent words from the students’ written answers to pre-course questions.

1. Relationship between human health and the environment

Many respondents expected the course would cover a global perspective on human, animal and environmental health, recognizing them as interdependent components of a single system:

“I expect to explore [ …] the planet's health as a determinant of health.”

Some cited climate change, pollution, and their effects on human health; others mentioned the challenges environmental disasters pose to the healthcare system, alongside public health policies to tackle them. Other expected topics were the concept of PH itself and solutions and approaches to mitigate human-induced degradation of the environment as individuals, future health professionals, and as a society.

Most respondents declared they would like the course to cover similar themes to those mentioned above, and some cited a multidisciplinary approach to health, antimicrobial resistance, and eco-anxiety.

Many respondents’ preliminary ideas about PH included a focus on the relationship between humans and the environment in general, while some, mentioned specific themes, such as the health effects of the climate crisis, and antimicrobial stewardship. Several mentioned a global and multidisciplinary approach to the complexity of population health, on both a national and international level, while others discussed an integration between ecology and medicine, and a concern for the planet and future generations.

2. International health emergencies

Several participants expected the course to address the healthcare response to international population emergencies in general, while others specifically mentioned migrations. Population migration was also mentioned among topics students wished the course would cover, along with the prevention and management of pandemics in general and regarding the COVID-19 pandemic. Several were interested in exploring the health consequences of current socio-political issues. Others included the role of international organizations and cooperation in managing health emergencies. Some respondents’ preliminary ideas about PH included international emergencies like war, pandemics, and hunger.

3. Characteristics and role of health systems

Several respondents emphasized the role of health systems, public health, prevention activities, and health promotion in terms of expectations for the course and the topics they wished to cover. Specifically, some expected the course to highlight the public health management of vulnerable populations, and others the reciprocal influences of the current global health scenario and the Italian health system.

Some respondents associated PH to a vision of a more responsive, fair, equitable health system, also focused on emergency preparedness and response. Others associated concepts of social fairness and access to care to interdisciplinarity.

“A non-sectorized, comprehensive model of health care that encompasses all fields of medical and non-medical knowledge to improve patient well-being.”

4. Broadening of knowledge and views

Some respondents expected the course to widen their knowledge and perspectives on the subject matter. Most of them wished to further expand on concepts related to PH only briefly mentioned in the medical school curriculum, and use this occasion to share knowledge amongst their fellow students.

“Delving into topics and issues that are little known and little covered in the university course, but in my opinion fundamental to the training of good physicians.”

The desire to expand perspectives and gain a deeper understanding of how PH impacts healthcare dynamics was similarly expressed in answers regarding the course's influence on their professional lives:

“Gaining a global perspective to meet the challenges of the future in health care.”

5. Positive professional impact of the course

Most respondents suggested a positive influence of the course on their awareness, future professional practice and career choices. They cited broadening of their horizons, gaining essential knowledge on health systems impact, and better understanding the human-environment relationship as reasons for this.

“I hope that it will enable me to approach my profession holistically: that is, it will enable me to approach clinical activity without neglecting the weaker segments of the social context in which I will work […]”

This positive attitude towards the course was also seen in those who did not wish to pursue a Public Health career.

“Although I do not see Public Health in my future career path, I think this course may provide me with an awareness of my actions as a physician, which are acts directed toward the patient, the community, and the planet. […] I hope to consider said medical acts differently than I would have thought, to safeguard ‘Planetary Health’, and perhaps modify them.”

A minority concluded that the course would have little or no impact on their careers, despite recognizing its role in broadening their personal knowledge, citing the lack of practical applications and a general disinterest in the field as primary reasons.

### 3.2 Post-course findings

#### 3.2.1 Quantitative findings

The assessment of satisfaction with each ES and the entire course are presented in Table 2, Table 4, respectively. In post-ES linear-scale questions, participants rated the presence of a participatory environment with mean scores ranging from 2·7 to 3·7 out of 4·0, and the suitability of teaching methods with mean scores ranging from 2·9 to 3·8 out of 4·0. No statistically significant differences were found between ESs for each item (*p* > 0·05), as shown in Table 3.

**Table 2:** Assessment of students’ satisfaction with each ES: mean and standard deviation of the scores assigned to each item (on a scale of 1–4) in the post-ES questionnaires.

Items	Mean score (SD) for ES1	Mean score (SD) for ES2	Mean score (SD) for ES3	Mean score (SD) for ES4
Overall satisfaction	3·7 (0·6)	3·1 (0·9)	3·2 (0·8)	3·5 (0·6)
Provision of inspiration and helpful resources for future academic and career decisions	3·4 (0·7)	3·0 (0·7)	3·1 (0·8)	3·4 (0·7)
Participatory environment	3·7 (0·5)	2·7 (1·0)	3·3 (0·7)	3·5 (1·3)
Lecturers' clarity of exposition	3·8 (0·7)	3·4 (0·7)	3·5 (0·7)	3·6 (0·6)
Suitability of teaching methods	3·8 (0·5)	2·9 (1·0)	3·3 (0·7)	3·5 (0·8)
Need to integrate the session topics in the medical curriculum	3·8 (0·5)	2·9 (1·0)	2·9 (1·0)	3·3 (1·0)

SD = Standard deviation.

**Table 3:** *p*-values from the statistical comparison of mean scores in Table 2, considering ES pairs.

Items	ES1 vs. ES2	ES1 vs. ES3	ES1 vs. ES4	ES2 vs. ES 3	ES2 vs. ES 4	ES3 vs. ES 4
Overall satisfaction	0.08	0.08	0.08	0.08	0.08	0.08
Provision of inspiration and helpful resources for future academic and career decisions	0.09	0.09	0.09	0.09	0.09	0.09
Participatory environment	0.09	0.09	0.09	0.09	0.09	0.09
Lecturers' clarity of exposition	0.57	0.57	0.57	0.57	0.57	0.57
Suitability of teaching methods	0.45	0.45	0.45	0.45	0.45	0.45
Need to integrate the session topics in the medical curriculum	0.20	0.20	0.20	0.20	0.20	0.20

**Table 4:** Assessment of students’ satisfaction with the entire course: mean and standard deviation of the scores assigned to each item (on a scale of 1–4) in the post-course questionnaires.

Items	Mean score (SD)
Overall satisfaction	3·2 (0·7)
Consistency of course contents with expectations	3·5 (0·6)
Provision of inspiration and helpful resources for future academic and career decisions	3·2 (0·6)
Participatory environment	3·2 (0·8)
Lecturers' clarity of exposition	3·5 (0·6)
Suitability of teaching methods	3·5 (0·6)
Relevance of the course to the medical education path	3·0 (0·8)
Need to integrate the course topics in the medical curriculum	3·4 (0·7)
Positive influence/impact of the course on one's future career	3·1 (0·9)

SD = Standard deviation.

#### 3.2.2 Qualitative findings (comments and suggestions)

Fourteen students answered the only open-ended question in the post-course questionnaires. Respondents shared a need for more detail on the practical and operational aspects, and for a more interactive learning experience, with shorter lecture-driven sessions, and increased opportunities for first-hand student activities and direct dialogue. Some expressed a desire to learn more about potential career paths in Public Health and a need to understand how to apply what they learned in the course to their future practice of medicine.

“In my opinion, the teaching mode should be more, if not entirely, interactive; in addition, special attention should be given to the more practical and concrete aspects of discourses that are often too theoretical [ …].”

## 4 Discussion

These results showed that the introduction of a PH elective course has been generally appreciated by the students. The quantitative analysis indicated a substantial pre-existing interest in PH topics as the primary motivation for enrolling in this elective, and upon completion it revealed high satisfaction regarding the teaching methods and course content. The qualitative analysis of the open-ended questions section identified five main themes emerging from the pre-course responses, some of which, along with their subtopics, aligned with those outlined in recently developed conceptual frameworks for PHE [3,[12], [13], [14]].

### 4.1 Relationship between human health and the environment

This theme, embodying the fundamental concept of PH, recurred across all students' written responses to the pre-course questionnaire, and interest in it was one of the main reasons for enrolling. This topic overlaps with those present in the various conceptual frameworks of PHE, e.g. “The Planetary Health education framework” of the Planetary Health Alliance (PHA) by Guzmán et al. and its domains “Interconnection within nature” and “The Anthropocene and health” [3]. Among the subtopics, the need for a holistic and multidisciplinary approach to health was frequently mentioned, and this is very relevant, given the prominence that transdisciplinarity has in the cross-cutting principles for PHE, as delineated by Stone et al. ^13^*,* and in the “Complexity and Systems Thinking” domain of the PHA framework [3].

### 4.2 International health emergencies

Several respondents mentioned this theme, indicating their awareness of the increasing complexity and interconnectivity of our world, and interest in it was also a popular reason for enrolling. Notably, the Canadian Federation of Medical Students Health and Environment Adaptive Response Task Force (CFMS HEART) incorporated disaster preparedness into the evidence-based Planetary Health Educational Competencies (PHEC) [14]. Moreover, respondents frequently mentioned the subtopic of international cooperation, which is pivotal to upholding principles of equity, justice, and sustainability [15]: thus it is worth noticing that Guzmán et al. consider “Equity and justice” as the fourth domain encapsulating the essence of PH knowledge, values, and practice [3].

### 4.3 Characteristics and role of health systems

Students showed positive attitudes to exploring and learning the salient aspects of the healthcare system and the preventive and health promotion functions of Public Health, and understanding interventions tailored to more disadvantaged populations' needs. In line with this, CFMS HEART considers training about “Marginalized and at-risk populations” among the PHEC [14]. Furthermore, the environmental sustainability of the healthcare system recurs in several responses, underscoring the students' awareness of healthcare systems’ responsibilities regarding significant environmental waste and contamination, as well as the pressing need to address these concerns [16].

### 4.4 Broadening of knowledge and views

Most students expected the course to broaden their knowledge and world views. This might be related to the common perception of Italian medical education as restrictive and extremely domain-specific. Furthermore, students worldwide have long reported that PHE was either completely missing or insufficient [6], suggesting that domain-specificity is an international issue. The broad participation to our course is thus in line with the willingness of a complete integration of climate change and related topics in medical curricula, as envisioned by the International Federation of Medical Student Associations (IFMSA) [17], to gain the potential to enrich many generations of future health professionals.

### 4.5 Impact of the course on working in the health sector

Most respondents expected that the course teachings would positively influence their career and professional practice, often associating this theme with one or more of the others. These associations reveal that respondents envisioned the medical profession as inextricably linked to higher-order concepts, recognizing the role of medical education within multiple complex systems. This aspect is also reflected in Guzmán et al., where systems thinking is indicated as fundamental for a better-structured PHE [3]. In the post-course questionnaires, respondents attributed mean scores of 3·2 and 3·1 out of 4·0 to the items “Provision of inspiration and helpful resources for future academic and career decisions” and “Positive influence/impact of the course on one's future career”, respectively. These results suggest that the course fulfilled the participants' expectations in this respect.

### 4.6 Generic comments, suggestions, and feedback

Feedback from participants was generally positive, according to both qualitative and quantitative findings. Their demands for a more interactive way of learning and for a focus on the practical aspects of the subjects covered emphasize the need to surpass traditional teaching approaches, as supported by a recent German survey, which showed that educators, students as educators and study deans believe that PHE should be student-oriented, use innovative and evidence-based competency-oriented methods of teaching and assessment, and teach transformative competencies including practical skills [18].

### 4.7 Relevance of the incorporation of PHE in the medical curriculum

This is a pilot course and marks the initial attempt to integrate PHE into the medical curriculum in Italy, albeit as an elective. As shown by post-course quantitative findings, participants agreed on the need to integrate the course topics in the medical curriculum, and on the relevance of the course to the medical education path. This assumes greater significance given the expanding scientific literature endorsing this integration [18,19], already supported by several organizations and institutions [[20], [21], [22], [23]]. Medical students have been advocating for it since the conceptualization of PHE in 2015, leading numerous initiatives globally, e.g., the 2018 statement by the IFMSA [6], CFMS HEART's proposed PHEC framework [14], and the Planetary Health Report Card (PHRC) [24].

Nevertheless, the incorporation and monitoring of PHE into medical curricula remains severely lacking, both globally and locally [6]. Italy, recognized as a hotspot for climate crisis along with the entire Mediterranean region, faces significant vulnerability [25], with two salient examples being the flood occurred in May 2023 in the Emilia-Romagna region in Northern Italy [25,26], and the upsurge of heat-related mortality rates [27]. Robust mitigation and adaptation measures should go along with long-term approaches based on widespread education and awareness initiatives, and on the comprehensive training of skilled professionals. Italy could be a good candidate to join the global PHRC community to facilitate the evaluation and enhancement of PHE in Italian medical degree programs, relying on the expressed interest and leadership capacity of local medical students and faculty [24]. In particular, teachers of clinical disciplines such as cardiology, pneumology and metabolic diseases can be inspired by these findings to integrate the role of environmental degradation in the onset of many diseases into their teaching. In addition, teaching on Planetary Health should be offered also in the early years of medical school, to involve future doctors from the start of their academic careers.

### 4.8 Strengths, limitations and future perspectives

To the best of our current knowledge, this study represents the first investigation conducted within an Italian context analyzing students’ perspectives on PH in the context of a PH course, and one of the first globally. Therefore, it could provide valuable insights for the effective design and implementation of PH courses in medical degree programs.

This study has some limitations. Firstly, the limited sample size, albeit sufficient for qualitative analysis, reduced the statistical power for quantitative analysis. Furthermore, since the course was elective, the sample was not representative of the entire fifth-year medical student population. Finally, the course did not systematically address PH, but explored only a limited range of its issues.

In future studies, expanding qualitative approaches, such as focus groups, could further enrich understanding of students' engagement with these issues. Moreover, they should aim for broader and more representative cohorts to strengthen quantitative analyses. Additionally, refining questionnaires to capture not only knowledge but also attitudes and behavioral intentions could provide deeper insights. More structured integration of PH topics into medical curricula—beyond elective courses—would also allow for longitudinal assessments of students’ evolving perspectives.

### 4.9 Conclusion

This study revealed considerable students’ sensitivity to the tight connection between Earth protection and the medical profession. Along with the international context of student activism around the planetary crisis, this gives hope that the next generation of physicians has both the willingness and the capacity to enact positive change in healthcare and beyond. This work contributes to the vital conversation about the structural integration of PHE into medical curricula, with a focus on customizing content and teaching methods to better engage students.

## Ethical committee approval

This study was compliant with the Local Ethical Committee Standards of the Faculty of Medicine and Surgery of Policlinico Universitario Agostino Gemelli IRCCS. It was approved and registered (Prot. N° 0012410/23 ID: 5676) and carried out in accordance with the Helsinki Declaration and EU Regulation 2016/679 (GDPR). The Ethical Committee foresaw the need for participant consent.

## Data availability statement

The data underlying this article will be shared on reasonable request to the corresponding author.

## Declaration of generative AI and AI-assisted technologies in the writing

During the preparation of this work the authors used DeepL Write to improve readability. After using this tool, the authors reviewed and edited the content as needed and take full responsibility for the content of the publication.

## Funding

This research did not receive any specific grant from funding agencies in the public, commercial, or not-for-profit sectors.

## Declaration of competing interest

The authors have no conflicts of interest to declare.

All co-authors have seen and agree with the contents of the manuscript and there is no financial interest to report. We certify that the submission is original work and is not under review at any other publication.
